# Praiseworthiness and Motivational Enhancement: ‘No Pain, No Praise’?

**DOI:** 10.1080/00048402.2019.1618883

**Published:** 2019-06-19

**Authors:** Hannah Maslen, Julian Savulescu, Carin Hunt

**Affiliations:** a University of Oxford; b King's College

**Keywords:** praiseworthiness, effort, commitment, achievement, motivational enhancement

## Abstract

The view that exertion of effort determines praiseworthiness for an achievement is implicit in ‘no pain, no praise’-style objections to biomedical enhancement. On such views, if enhancements were to reduce the need for effort, agents would be less praiseworthy. Motivational enhancement would appear to be the most problematic in this respect, given that increased motivation reduces the need for agents to rally themselves and to exert effort in activity. We use the prospect of motivational enhancement to re-examine the grounds of praiseworthiness for achievements. We consider the place of effort amongst the grounds for praise, whether effort exhausts these grounds, and how they can be better specified. We argue that praiseworthiness depends on (i) the voluntariness and strength of the agent’s committed pursuit of a valuable end (E), (ii) the costliness of the committed pursuit of E, and (iii) the value of E. Effort is just one cost amongst many, and costs of activities can be traded-off. Motivational enhancement reduces the praise due to an agent only when it reduces the net cost to the agent (without strengthening the voluntary commitment). We emphasize the importance of a diachronic perspective on active agency for praiseworthiness, to include training, prior planning, and deliberate strategies to overcome weakness of will, even where this reduces the need for effort.

## 1. Introduction

Yet in those areas of human life in which excellence has until now been achieved only by discipline and effort, the attainment of those achievements by means of drugs, genetic engineering, or implanted devices looks to be ‘cheating’ or ‘cheap’. We believe—or until only yesterday believed—that people should work hard for their achievements. ‘Nothing good comes easily.’ [Kass 2003: 21]

Because they act directly on the human body and mind, biotechnological enhancements tempt us to shirk individual striving and struggle. [Fox 2005: 1150]

Imagine two agents training for a marathon. The first, Jacek, does not enjoy exercise, finding training a huge effort. Nonetheless, he forces himself to train regularly, and on the day of the marathon achieves an admirable time of 2:58 hours. The second, Maurice, does not enjoy exercise either, and similarly finds training a huge effort. Maurice, however, takes a motivational enhancer each time that he has training. As a consequence, he finds training much less burdensome than he otherwise would, enabling him to engage in it more often and for longer each time. On the day of the marathon, Maurice achieves the same admirable time, 2:58hrs, as Jacek does. How does the use of a motivational enhancer affect the amount of praise that we think is due to Maurice?

The philosophical literature on the ethics of biomedical enhancement has focused principally on enhancement of cognitive or physical capacities—memory enhancement, blood doping, and so on. A common objection to the use of biomedical enhancements is that their use reduces the amount of praise that the agent deserves for his achievement—the intellectual or athletic feat, for example. Such objections tend to rely on the view that moral value resides predominantly or entirely in the *effort*—particularly, *psychologically aversive effort—*that the agent expends, and that agents are praiseworthy as a result of their effortful production of something of value. Call this argument the ‘no pain, no praise’ objection.

There are a number of counter-arguments to the ‘no pain, no praise’ objection. Even if one assumes that effort is the basis for praise (and this paper will argue that this view is overly simplistic), many cognitive and physical enhancements do not remove the need for effort and so would not affect the agent’s praiseworthiness [Greely 2007; Maslen et al. 2014]. Others have argued that, even though enhancements might affect the ‘nature’ or ‘richness’ of a particular activity performed under their influence, these need not undermine the value of the new or augmented activity thereby undertaken [Schermer 2008; Santoni de Sio et al. 2016]. Further, on some conceptions of collaborative authorship, it has been argued that we should shift the locus of praise and blame for particular achievements from individual creators to the ultimate products of their efforts [Goodman 2010]. On such a conception, enhancement technologies could be construed as a ‘collaborator’ in such achievements.

We are in broad agreement with these existing counterarguments to the ‘no pain, no praise’ objection and the corresponding claim that the use of such technologies to improve capacities does not necessarily strip the agent of her grounds for praise. Rather, it seems that what agents do with their capacities—how they work with them—instead determines the praise due to an agent.

However, in addition to cognitive and physical enhancements, it may also be possible to enhance the agent’s will or drive via biomedical means; we shall term this ‘motivational enhancement’. If motivational enhancement reduced the need for effort, the concerns encapsulated in the quotations above would be brought into sharper relief. It is not clear that the above responses to the ‘no pain, no praise’ objection are applicable in the case of motivational enhancements. Such enhancements might be understood to reduce the need for effort in a way that cognitive and physical enhancements do not, and to play a more significant ‘collaborative’ role in achieving the goal in question, reducing the agent’s own contribution to the achievement. Although commentators are right to argue that there is ‘nothing *wrong* with shortcuts that enable us to reach certain results without pain, suffering or hard work’ [Schermer 2008: 363, emphasis added], we should attend directly to the implications of enhanced motivation for agents’ praiseworthiness.

We examine whether motivational enhancement would reduce the agent’s praiseworthiness as a consequence of reducing the amount of effort that he exerts. We argue that effort is not the fundamental ground for praise for achievements. Rather, an assessment of praiseworthiness should involve a diachronic perspective on the *strength* of an agent’s *voluntary* and *costly* commitment to pursuing *valuable* activities and achievements. We conclude that investment of effort constitutes just one type of opportunity cost relevant to assessments of praise. As a sketch, our account shows that the intuition that Maurice is less praiseworthy than Jacek (above) would be correct if all else were exactly equal. However, Maurice may not be less praiseworthy if he incurs alternative costs, or if taking the motivational enhancer underwrites his commitment to achieving the admirable time.

Our paper has important implications for interpersonal and formal practices of praising, for the legitimacy of social and (perceived) moral disincentives to enhancement use, and also for our philosophical understanding of what grounds praise more generally. We shall begin by situating our discussion in the context of existing literature on effort and desert.

## 2. Capacities, Motivation, and Effort

We have drawn a distinction between capacities and motivation as discrete targets of enhancement. It is worth emphasizing why this distinction is important for considerations of praiseworthiness, as well as for clarifying the relationship between motivation and effort.

The value of effort and its relevance to desert has been discussed in relation to the just distribution of economic benefits, such as income. In broad strokes, theorists’ views predominantly align with either (i) the claim that productivity (of social product) is the basis on which agents deserve economic benefits, or (ii) the claim that exertion of effort (towards social product) is the relevant distributive ground [Lamont 1994, 1995]. Although our discussion concerns deserved praise (and not necessarily any economic benefit), this literature is instructive.

Those who endorse claim (ii), identifying effort as the primary desert base for jobs or economic goods, often do so because alternative grounds for desert leave too much room for the influence of undeserved natural talents (what we have called capacities), which are not distributed equally (for example, Wasserstrom [1976]; Daniels [1978]; Miller [1996]). According to this justification of (ii), the level of effort that we put into a given endeavour, unlike natural talents, appears to be more ‘up to us’, and so it should be the principal determinant of what remuneration we deserve. Although some have argued that the capacity for exerting effort is similarly unjustly distributed [Rawls 1971], we follow a number of authors in our contention that exertion of effort is under agents’ control to a greater extent than their natural talents are (see for example, Sher [1979]). Any differences in propensity to exert effort will not bear on limits of performance in the same way as other capacities do, because of the possibility of employing ‘sheer force of will’. An agent’s inability to run a race quickly because of his short legs is not something over which he has any control. In contrast, his running as fast as his short legs can carry him, despite disinclination towards exerting effort, appears to be something over which he can exert some degree of control.

The thought that agents are to some degree responsible for the effort that they expend in exercising their capacities may explain the apparent intuitive appeal of the claim that effort and exertion are the central grounds for praise. While ‘it needn’t be that the foundations underlying desert are themselves deserved, all the way down’, effort, and the will that it reflects, can be understood to act as the mediator between natural talents and praise [Nozick 1974: 225].

We should clarify the relationship between effort and motivation, since the two are related but distinct. Motivation is a tendency *towards* action—a psychological disposition—whilst exertion of effort, producing aversive psychological features, occurs *in acting*. Whilst being motivated to carry out a particular act might be relevant to the overall moral assessment of an agent when they act according to some moral theories (for example, virtue theories), praise is usually seen as the appropriate response to engaging in effortful action, rather than merely to a state of being motivated. Being highly motivated will in many cases make engaging in action less effortful, and low motivation requires exertion of more effort on the part of the agent to engage in action.

We focus on biotechnological increases to motivation that may serve to reduce the need for effort. Empirical work on pharmaceutical enhancements has considered the consequences of enhancing motivation, as distinct from an agent’s capacities. Ilieva and Farah [2013a] argue that the assumption that stimulants such as amphetamine and methylphenidate (for example, Ritalin) are effective cognitive enhancers—boosting the attention and executive function of normal individuals—has been brought into question by recent evidence. The evidence they present suggests that many drugs thought to act on cognition in fact have only a limited direct effect on it, whilst their effects on motivation are significant: they provide a useful ‘*non-cognitive* advantage’ [Ilieva and Farah 2013b: 1]. They cite the following research participant’s remark as illustrative of the experience of this distinction: ‘Adderall doesn't necessarily make you smarter […], the main benefit, really, is that on it, I don’t mind doing work’ [ibid.: 3]. The need for effort is reduced.

While the scientific basis of the mode of action of these drugs is far from settled, in the remainder of this paper we address whether motivational enhancement would reduce an agent’s praiseworthiness for her achievements.^1^ We first examine more precisely what motivational enhancement could consist in.

## 3. Intrinsic and Extrinsic Motivation

Let’s begin by drawing a distinction between intrinsic and extrinsic motivation. Although a mixture of both will often sustain an agent’s pursuit of long-term projects, putative enhancers have been identified as having effects more on one or on the other, and we consider both types of motivation.^2^ Intrinsic motivation, we will show, generates the more interesting *prima facie* theoretical problems for motivational enhancement, and for maintaining grounds for praise.

Intrinsic motivation is the psychological mechanism and accompanying phenomenological experience of finding a particular activity inherently rewarding, thus conferring on the agent a propensity to pursue it. The more that an agent is intrinsically motivated to perform some action, such as to study, the less psychologically burdensome it is for him. The agent is motivated because he enjoys the task. Intrinsic motivation thus ‘exists in the nexus between a person and a task’ [Ryan and Deci 2000: 56]. While the agent expends energy when intrinsically motivated, this does not constitute exertion of effort *per se*. This is because exerting effort is characterized by an aversive phenomenology, and the expenditure of energy in the pursuit of something that one is intrinsically motivated to do is not experienced as aversive. Intrinsic motivation reduces the need for effort since expending energy on the particular task is inherently rewarding to the agent when he is intrinsically motivated.

In contrast, extrinsic motivation spurs the agent on when he is ‘doing something because it leads to a separable outcome’: the agent focuses on the reward that this outcome is expected to deliver [Ryan and Deci 2000: 60]. The agent experiences this type of motivation as being spurred on by anticipated rewards *despite* the task not being enjoyable. This kind of motivation engages self-regulation, which is what deters agents from slowing their running pace when tired and in pain, or from checking social media while working at something they find tedious. That is, it deters people from choosing small immediate rewards over delayed larger ones [Berkman et al. 2011; Crockett et al. 2013].

Increasing intrinsic motivation is one mechanism of motivational enhancement. Studies investigating the effects of Modafinil on performance have concluded that it increases subjective task enjoyment [Müller et al. 2004; Ilieva and Farah 2013b]. Enhancement of extrinsic motivation is also possible. Results of a double-blind placebo-controlled study suggest that amphetamine increases willingness to endure effort and probabilistic costs to obtain monetary reward. This is thought to be due to its effect on dopaminergic mechanisms in the brain [Wardle et al. 2011]. Amphetamines have been widely used in cycling and baseball as performance enhancers. Similarly, transcranial electrical stimulation of the brain has recently been shown to improve cyclists’ time to exhaustion [Angius et al. 2016]. Enhancing extrinsic motivation appears to reduce the need for effort as it increases resolve, but it does not remove the need for effort.

Before we examine the implications for agents’ praiseworthiness, we first distinguish between praising agents (for producing products) and independently valuing the product of their agency.

## 4. Praising Agents, Valuing Products

In order to assess the amount of praise due to an agent, we must demarcate what is relevant. We can distinguish the moral assessment of the agent (for producing the product) from an independent (non-moral) assessment of the value of the product. The former involves praise of the agent, the latter something like appreciation of, or wonderment at, the product. Praiseworthiness does not simply track the value of the product (which can be admired or wondered at, regardless of the agent’s specific contribution to its production). This points to the central importance of the agent’s activity for praise. However, as we will argue below, the value of the product is not irrelevant to praise, and intuitions regarding praise for seemingly ‘effortless’ valuable performance motivate the idea that momentary effort does not exhaust the morally relevant features of the agent’s contribution.

It seems undeniable that we are often focused on our positive attitudes towards great products or performances—artworks, technological advances, sporting feats—and that these attitudes tend to colour our assessments of the agents that produced them. However, our positive attitudes towards products and performances can still occur when there is no agent (perhaps we appreciate a beautiful computer-generated artwork) or when there is a non-moral agent (perhaps we wonder at a cheetah’s sprint). Whilst these positive attitudes to products or performances are an appropriate response to value, they should not eclipse or substitute for the moral assessment of agents that produced them.

When we turn our attention to agents, we can make comparative assessments of praiseworthiness for producing *equally valuable* products. There are factors that affect this assessment which are not exhausted by the value of the product. Two agents may differ in the extent to which they ‘strove and struggled’ in order to produce the equally valuable products—more specifically, in the amount of effort that they exerted in the products’ production. The value of the product does not determine this amount.

Despite the distinction between praising the agent and valuing the product, it seems too strong to conclude that many agents whose products we appreciate will be rendered significantly less praiseworthy given their apparent effortlessness—the lightning demonstration of a mathematical solution, a great shot by Roger Federer, or Usain Bolt smiling at the cameras as he wins an Olympic final with seeming ease. There still seems much to praise in these cases. However, this should not prompt us to revert to the view that the value of the product determines the agent’s praiseworthiness. Rather, we argue that the intuition to resist this conclusion (that momentarily effortless displays are not praiseworthy) is actually consistent with the view that praise is grounded by the agent’s active contribution to the product. Indeed, we argue that illuminating this prompts a more nuanced analysis of the grounds for praise.

In many cases of effortless performance, the apparent effortlessness will not detract from the praise due to the agent if the achievement involves *diachronic* exertion of effort—that is, the exertion of effort across time. The agent’s significant contribution is made over time. There are very few people, if any, for whom great creative, intellectual, or athletic feats have not involved effort at some point. Reaching the point where one can perform a task effortlessly often involves a substantial amount of prior training, learning, and practice, including the effort involved in deferring gratification in order to pursue something difficult. This diachronic perspective on effort demonstrates that the apparently easy performance in fact constitutes ‘effortful effortlessness’, where the effort was invested at an earlier time. Further, where agents seem to effortlessly produce things of value, we can additionally point to their *choice* and *commitment* to pursue these valuable products as part of the agent’s active contribution to the product. That the product bears independent (non-moral) value is relevant to (but not solely determinative of) the praise due to the agent.

Two preliminary conclusions can be reached. (i) Although the value of the product does not directly determine praise, its value will be relevant to the praiseworthiness of the agent’s choice to pursue it. And (ii) the morally relevant aspects of the agent’s contribution will include but not be exhausted by their momentary exertion of effort; choice, commitment, and training over time will also be amongst the grounds for praise.

Taking these preliminary conclusions as our starting-point, we now offer an account of the grounds of praise for achievements.

## 5. The Grounds for Praise for Achievements

*Contra* the bioconservative view captured at the beginning of this paper, it is not only or even principally the aversive experience of effort—the striving and struggling—that confers moral value and grounds praise. Agents can justifiably be praised for the choices that they make, and for their commitment to them. Praiseworthy commitment involves considerable opportunity costs, incurred by investing time, energy, wellbeing, and other limited resources, as well as explicit priority-setting by the agent. Effort is another such cost and is often a good proxy for the strength of this commitment. However, it is the choice and costly commitment to pursue a valuable achievement that is fundamentally praiseworthy. We now defend and formalise this position.^3^

Our position broadly aligns with Aristotle’s discussion of agency and praiseworthiness in Book III of the *Nicomachean Ethics*, and we take this as a starting-point.^4^ Here, Aristotle considers what it is for an agent to be the author of his actions. He argues that a voluntary act is an act the origin of which lies in the agent. However, the voluntary nature of an act is not all that is relevant when morally evaluating an agent for performing it. Aristotle argues that choice, a species of voluntary act, is more intimately connected with virtue, and hence with praiseworthiness. He defines chosen action as voluntary action preceded by deliberation, since rational choice involves reasoning and thought [1112^a^14]. Rational choice, he says, ‘will be deliberative desire for things in our power; for, when we have decided on the basis of deliberation, we desire in accordance with our deliberation’ [1113^a^11–14].

We suggest that this deliberate, concerted nature of choosing and fixing our desire is central to the grounds of moral assessment for achievements, and constitutes what we will call the archetype of ‘active’ agency—agency that issues from the agent in the form of repeated chosen acts. Further, the more that an act (or succession of acts) requires some sacrifice or investment (in terms of energy, time, wellbeing, material resources, and indeed effort), the more concerted is the choice to perform the act(s). It is this aspect of active agency—the agent’s costly commitment—that is of primary relevance for praise. Although effortful action may reveal or constitute costly commitment in many particular instances, it is not the only cost involved in commitment, nor the only locus of moral value. Further, agents can trade off effort for alternative relevant costs, as well as reinforce their commitment by strategically reducing the need for effort. We defend now these two claims.

*Effort is one relevant cost amongst many*: Effort is just one cost amongst many that in sum determine the costliness of an agent’s commitment. For example, an agent who exerts substantial effort to paint a masterpiece over the course of a week bears different costs to the agent who exerts much less effort but more time to completing the masterpiece, incurring greater opportunity costs. Praise is deserved when costly commitment is present, even if these costs do not include effort.^5^

*Strategic effort reduction can be praiseworthy*: The importance of commitment (sometimes over effort) is revealed in the praiseworthy dimensions of self-control strategies, some of which in fact make things easier for the agent to achieve their goal. Self-control requires understanding one’s limitations and setting strategies and procedures to externally control one’s weakness of will—for example, setting a timetable for studying or training, or removing distracting temptations.^6^ Notice that these are designed to increase time spent efficiently on a task. Such strategies may mean that performance at a time is less effortful, even though greater time is spent on the task, but this is due to the voluntary prior planning of the agent and the employment of pre-commitment contracts, which are praiseworthy *precisely because* they demonstrate commitment and they serve to structure the agent’s activity towards achievement of the valuable goal. Such consciously employed strategies to overcome weakness of will (counteracting an extant or anticipated lack of a control) are to be praised. Such a person may even enjoy the performance of a task that would have been more effortful without the effort-reduction strategies, but this kind of structured enjoyment is an appropriate object of praise. It requires conscious agency to plan and execute, and a level of strength of will to employ.

Thus, although effort plays a role in the grounds of praise, praiseworthiness does not depend only or even necessarily on the ‘striving and struggling’ emphasized in the bioethical literature. Instead, the amount of praise due to an agent is principally determined by the strength of costly commitment invested in pursuit of a valuable end.

More formally, the notion of costly commitment, which varies in strength, can be described as follows:

**Costly commitment to valuable end *E***: voluntary, sustained prioritization of *E* through making successive *E*-directed choices and allocating limited, depletable resources (effort, time, energy, material goods, well-being, other opportunities, etc.) to the pursuit of *E*.

There are three morally relevant dimensions to this description:

(1) the *voluntariness* of the agent’s committed pursuit of *E*,

(2) the *costliness* of the committed pursuit of *E*, and

(3) the *value* of *E*.

Further, (4) the *strength* of the agent’s commitment—the *extent to which* the agent prioritizes the sustained pursuit of *E*—will also be relevant.

The first dimension relates to the *voluntariness* of the pursuit of the valuable end. In order to be a candidate for praise at all, the agent must be responsible for the decisions and actions directed towards achieving the valuable end. Its pursuit must have been voluntary. We might correspondingly think of voluntariness as a threshold necessary condition for praise for achievements. Although we do not have space to provide a full account of the conditions for voluntariness, or to suggest where exactly the threshold should be set, we hope that an intuitive sketch will suffice to defend the claim that voluntariness will be a necessary threshold condition for praise. Voluntariness might be compromised by either external or internal factors. If, for example, an agent is coerced or compelled by another agent into engaging in costly activity towards a valuable end, then such activity may not be sufficiently voluntary to render the agent a subject of praise. For example, some offenders carrying out community service *against their will* are not candidates for praise for their ‘achievements’, even when their activity is valuable and costly. Further, internal factors could render activity insufficiently voluntary for praise when, for example, individuals engage in activity *compulsively*, without reflective endorsement. Some obsessive-compulsive behaviours would meet this description. The voluntariness threshold for praiseworthiness is likely to be low—simply the requirement that the agent chooses to engage in the activity. In practice, most activity will meet this threshold. We include voluntariness for completeness of the account. The potential for substantial differences in praiseworthiness will hinge on the remaining three factors.

Praise for voluntary committed pursuit of *E* will be sensitive to the *costliness* and *strength* of the commitment (namely, the expected quantum of sacrifice, and the agent’s prioritization of *E*), and the *value* of *E*. Although costliness and strength of commitment will be related, our discussion of strategic effort-reduction above showed that an agent’s strategic reduction of costliness could sometimes demonstrate strong commitment, due to concerted prioritization of *E*. In general, the costlier and stronger the commitment, and the more valuable the end pursued, the more praise the agent deserves. Costliness tracks expected costs—the probability × magnitude of disvalue—rather than absolute costs.

The amount of praise due to an agent for an achievement will, we suggest, be a *multiplicative function* of costliness plus strength of the commitment and the value of the end pursued. Pursuit of ends with no or little value will not earn great praise, no matter how strong or costly the agent’s commitment to them. For example, an agent’s very strong costly commitment in pursuit of an end of no value (for instance, counting blades of grass [Rawls 2009: 380]) should not earn her much if any praise. Such an agent has ‘wasted’ her time, energy, and other resources on doing something with little or no value. Furthermore, an agent’s very strong costly commitment to an immoral end (represented as having negative value) will not only fail to earn her praise, but would plausibly attract greater blame, the more costly and strong the commitment.

Praise should not be too dependent on luck. Not all agents will be capable of achieving ends of the highest objective value. Agents should not be precluded from ever being particularly praiseworthy as a consequence of their limited capacities. Thus, the value assigned to the end should be corrected for the value of that end *relative to* the range of valuable achievements that the particular agent is able (due to capacities and circumstance) to pursue. Just as we would direct agents away from pursuing valueless ends, we would direct agents away from pursuing ends that they are incapable of achieving, regardless of the objective value. If we want to minimize the contribution of luck to praiseworthiness, we would need to allow that agents could be maximally praiseworthy if they maximally commit to pursuing the most valuable ends that they are capable of achieving.

A full account would require an account of the value of different ends. We take it as intuitive that ends do differ in their value: pursuing an advance in medicine is a more valuable end than counting blades of grass is, and an end such as inflicting pain has disvalue.

## 6. Motivational Enhancement and Praise

To see whether and how motivational enhancement affects praiseworthiness, we must consider its implications for the agent’s choosing of, and costly commitment to, the valuable end (that is, implications for voluntariness, strength, and costliness). For the sake of analysis, we keep fixed the value of the end. We consider first the costliness of the commitment.

Although we have argued that effort is not necessary for praise, expending effort is one of a number of possible costs involved in seeing through one’s commitment to a valuable end. The drug Modafinil has been reported as increasing the intrinsic rewardingness of engaging in some cognitive tasks. Such an effect would plausibly reduce the need for effort, although the same amount of time and energy might still be invested. Another example might be the use of painkillers, nonsteroidal anti-inflammatory drugs, and local anaesthetics in sport, which make the engagement in the activity less painful and thus less effortful. Such interventions may be indirectly motivational as they make engagement in the activity relatively more rewarding (less aversive). If a motivational enhancer simply reduced the effort required to achieve the same goal, the commitment is rendered less costly for the agent. This would straightforwardly reduce the amount of praise due to the agent.

However, it is possible and even likely that the use of motivational enhancement will generate, or at least be accompanied by, alternative costs, such that net costliness remains equivalent or greater. For example, if it were the case that an agent were able, as a result of enhancing her motivation, to invest more time and energy in pursuing the valuable end, then she would have traded the bearing of one type of cost (exertion of effort) for others, such as expected risk of injury, time devoted to activity, etc. In such cases, the overall expected costliness of her commitment may remain the same or increase. Further, some motivational enhancers may directly impose costs on the agent. If the use of Modafinil is expected to disrupt the agent’s ability to sleep properly and/or deplete the energy available to her on subsequent days, then she bears an alternative cost. Similarly, if the athlete who takes pain killers, nonsteroidal anti-inflammatory drugs, and local anaesthetics knowingly risks greater injury as a result of increased physical exertion, then she bears an alternative cost, increasing praiseworthiness.

Net cost reduction would reduce the amount of praise due to that agent if we consider only the *costliness* of the commitment. However, we argued above that the *strength* of the agent’s commitment was also a morally relevant consideration. The strength of the agent’s pursuit of the achievement is a function of her controlled prioritization: this, as we suggested, is indeed central to what it means to be committed. How does motivational enhancement affect this consideration?

Irrespective of the way in which the use of a motivational enhancer shifts the costs involved in seeing through the commitment, it can be seen as part of making and taking seriously the commitment itself, strengthening it. In taking steps to better ensure the achievement of the valuable end, the agent demonstrates the priority that she gives to it and her intention to see it through. Agents can, of course, be maximally committed to an end without using motivational enhancement, but full commitment involves planning and prioritizing of some sort: motivational enhancement can be a species of such priority-setting, which is itself praiseworthy. Strengthening one’s commitment in this way could therefore counterweigh any net decrease in cost.

Instructively, notice that motivational enhancement would have different consequences for praiseworthiness if it were not the agent’s choice to use it. Imagine members of a sports team who are given a motivational enhancer in their daily breakfast by their coach. Whilst it is still the case (we assume) that they participate voluntarily in their training, the use of motivational enhancement here would not be reflective of the *strength* of their commitment (nor would any costs of enhancement be expected by the athletes). The agent who is dosed by their coach is not choosing to use the motivational enhancement strategically. It does not underscore her commitment to pursuit of the valuable end.

The remaining consideration is therefore voluntariness. Enhancement of intrinsic motivation would be problematic for praise if it were to render the agent ‘passive’, in a way similar to the coerced or compelled agent. This would occur if taking the enhancer hijacked agency in a way that rendered involuntary the choices and commitments of the agent. As with the offenders carrying out community service against their will, we would reduce the amount of praise bestowed on the agent who only passively sustained activity towards an achievement. This extreme possibility would affect the praise due to the agent for that achievement as a result of insufficient voluntariness. Whilst conceptually possible, the putative motivational enhancers noted in this paper are unlikely to render acts sufficiently involuntary and agents sufficiently passive to invalidate praise.

Motivational enhancement, then, can reduce, maintain, or increase an agent’s praiseworthiness for achieving a valuable end, depending on the way in which the enhancement affects the overall costliness and strength of the agent’s commitment.

So far, we have considered how motivational enhancement might affect the praiseworthiness of an agent, keeping the end she pursues fixed. We will now see how this analysis bears on examples of agents engaging in exercise to achieve a healthy weight. We then consider how the range of ends available to an agent can also have implications for what is most praiseworthy, and how motivational enhancement might ‘free up’ an agent to pursue a greater number of valuable ends, thus increasing her overall praiseworthiness, even if individual achievements were consequently less costly.

## 7. A Case Study: Motivational Enhancement, Responsibility, and Praiseworthiness for Healthy Weight

Compare three examples:

Karl likes to be sedentary and rarely does any exercise. Luckily for him, he was born with a fast metabolism and maintains a healthy BMI without even thinking about whether this is something that he wants to do.

Berit loves exercising and has always found it an enjoyable pastime, training five days a week. She is happy that this has the consequence of maintaining her healthy BMI.

Jacek hates exercise but wants to maintain a healthy BMI. He forces himself to go to the gym a couple of times each week, and this keeps his BMI within the healthy range.

Karl’s healthy BMI is the result of luck. There is a sense in which his healthy BMI is relatively unavoidable, and he does not intend to maintain it—it happens to him. Karl, it seems, is least praiseworthy for his healthy BMI, as well as being the least responsible. Berit also has some good luck in her enjoyment of exercise: she is intrinsically motivated. She intends to maintain a healthy BMI, but that she succeeds in doing so is a happy by-product of doing something that she enjoys. Although she could have spent her time on other activities, she does not have to expend much effort exerting control over her decision and commitment to spending time exercising. This suggests that the costs for Berit are higher than for Karl (she still invests time, energy, and incurs opportunity costs) but lower than for Jacek (who also bears the cost of exerting effort). It is also possible that Berit’s *commitment* is less concerted than Jacek’s, even though her engagement in exercise is as voluntary.

Although it is clear that both Berit and Jacek are more praiseworthy than Karl, it would be too quick to conclude that Berit is less praiseworthy than Jacek, despite the lower costs to her in terms of effort and the additional evidence of commitment on the part of Jacek. Our account has demonstrated that there are further morally relevant factors: Berit may spend more time due to her intrinsic motivation to exercise, and therefore incur extra opportunity costs relative to Jacek. Further, Berit’s being motivated could be the consequence of a history of sustained prioritization and cultivated enjoyment. The question of who is more praiseworthy would require a comprehensive assessment of all of these morally relevant factors, which could be stipulated for the sake of analysis to yield different outcomes: in some, Berit will be the most praiseworthy; in others, Jacek will be.

However, the complexity of the analysis does not speak against our account. That there are many morally relevant features, which can vary in combination and strength is, we believe, a plausible outcome for moral evaluations of praiseworthiness, and we have outlined and defended these factors at a level of some generality. Further, the different possible outcomes for Berit and Jacek, depending on the exact features of their costs, commitments, and choices over time resolve a tension between broadly Kantian and Aristotelian views. On our account, acting ‘out of duty’ in the face of costs is praiseworthy (Kantian), as are efforts to reduce the costs of an activity by cultivating motivation towards and enjoyment of this activity (Aristotelian). Indeed, Aristotle’s view on choice and praiseworthiness, discussed above, which included the concept of ‘*deliberative* desire’, aligns with an outcome according to which both deliberation and cultivated desire have moral value.

Now consider a fourth agent, Maurice, who also maintains a healthy BMI. However, this is not as a result of an unusually fast metabolism, finding exercise inherently rewarding, or forcing himself to endure burdensome exercise. Instead, Maurice takes a drug, ephedrine, which reduces perceived exertion [Williams et al. 2008; Haller et al. 2008]. Maurice finds exercise much less burdensome than he otherwise would, enabling him to engage in it more often and for longer each time. As a result, he maintains a healthy BMI.

Maurice’s commitment to exercise is underscored by taking ephedrine. How praiseworthy he is, relative to the others, will depend on whether he substitutes effort for other costs (for example, risk of side effects of the drug, injury, longer training hours, etc.) and on how the strength of commitment evident in his strategic use of enhancers weighs against any net reduction to the costs that he bears.

In taking a motivational enhancer, Maurice makes an additional choice in line with his commitment to maintain a healthy BMI. In terms of opportunity costs, Maurice sacrifices just as much time and energy as Berit and Jacek do. He does not exert as much effort as Jacek does, and so preserves more of this limited resource, but his commitment to maintain a healthy BMI may be at least as strong as Jacek's. Assuming that the effect of the enhancer does not compel him to train, Maurice could be as praiseworthy as Berit and Jacek are.

## 8. Global Praiseworthiness and Counterfactuals

We have so far restricted our analysis to discrete, fixed, valuable ends. We might think of these as local assessments of praiseworthiness, with respect to one specific achievement. How much *global* praise is due to the agent depends also on the relevant counterfactuals. Alternative valuable ends that an agent could have pursued are relevant. Imagine that John could exert his effort, time, and energy to achieving one highly valuable end—for example developing a vaccine for an infectious disease. However, he could alternatively have used the same total effort, time, and energy to develop three vaccines for three different infectious diseases. Although John deserves praise for developing the single vaccine, he would be more praiseworthy for developing the three vaccines for three infectious diseases, even though the commitment to developing each of these was individually less costly than the costliness of the commitment to developing the single vaccine. This shows that, when we think about the praiseworthiness of an achievement, we can make an assessment of praiseworthiness for that achievement in isolation, but we make a more accurate assessment of the global praiseworthiness of the agent if we consider the set of valuable ends open to him. Whether and how an agent makes use of the resources that are ‘freed up’ by enhancement will be relevant.

An additional consideration relates to the recklessness of *not* reducing the cost of the commitment where possible. Imagine that John could possibly develop effective treatment for a new deadly virus by doing all of the calculations by hand, which would take a huge amount of cognitive effort that he wasn’t sure he would be able to exert. Or, alternatively, imagine that John could use a new calculation tool to assist with his work, which would drastically reduce the effort that he needed to exert, thereby making commitment to developing the vaccine less costly. If John choses the more costly option, he risks the vaccine’s not being developed, and hence risks hundreds of lives. We would think this choice reckless and wasteful (once he has adopted the goal) and, whilst still praiseworthy in the event of success, less praiseworthy than using the calculation tool to ensure success.

Thus, how an agent distributes their limited resources (effort, energy, time, etc.) can bear on their global praiseworthiness. Although we are discussing praiseworthiness in the realm of supererogatory acts, we can still assess an agent as *more* praiseworthy when they distribute their limited resources so as to achieve a greater number of valuable ends with greater certainty. Costs should not be recklessly or gratuitously increased.

These considerations bear on the implications for motivational enhancement. If, for an agent like Maurice, taking a motivational enhancer substantially increases the chance that he will complete his exercise regime, then he will be more praiseworthy for taking it than for relying on the unlikely prospect that he will summon enough effort to complete the regime. So, even where use of an enhancer reduces the overall costliness of the commitment, the bearing that this has on praise must be balanced against the chance of failure through lack of motivation, and in light of any additional valuable ends that he was consequently able to (and did) pursue.

## 9. Conclusion

The view that aversive effort grounds praise is not entirely wrong, but it requires greater nuance: exertion of effort is not the sole locus of moral value.

Praiseworthiness for achievements is determined by the total costs that an agent invests or risks in pursuing an end of a certain value, as well as the strength of their commitment, demonstrated by concerted prioritization of this end.

Our account emphasizes the importance of a diachronic perspective to praiseworthiness, taking into account prior effort, training, and self-control strategies, such strategies themselves being partly constitutive of commitment, even where they reduce the need for effort.

Wittgenstein said, ‘the measure of a man’s greatness would be in terms of what his work cost him’ [Engel 1970: 486]. Wittgenstein is half-right. What matters for praise is not only cost, but also commitment to worthwhile ends. And effort is only one type of cost.

## Footnotes

1 Kjærsgaard [2015] has addressed some of the ethically problematic dimensions of motivational enhancement. Although his paper makes a valuable contribution to the discussion of motivational enhancement, his concerns focus on the prudential, rather than moral, disvalue of motivational enhancement. He considers the possibility that use of motivational enhancement might lead the agent to neglect the development of stamina. He also questions the effects that motivational enhancement would have on the agent’s authenticity (such that the agent does not know whether he is really interested in the project that he is artificially motivated to pursue).

2 Indeed, extrinsic motivation, such as the prospect of recognition by others, may become internalized such that the agent becomes intrinsically motivated to pursue subsequent similar achievements.

3 We do not develop an account of achievements here. For one recent attempt see Bradford [2015].

4 Translations of Aristotle’s text are from Crisp [2000].

5 The claim that various costs are relevant to praise-due has parallels with the view that economic benefits are deserved as a matter of compensation for the disutility that the agent accrues in producing social product (For a version of this view, see Ake [1975] and, for criticism, see Miller [1997].) Whilst this has some limited appeal in the context of the employment market, we do not think that it is appropriate to construe praise as a form of compensation—offsetting loss. Rather, it is a moral evaluation and recognition of the agent’s having performed a morally good act.

6 This point has similarities with Sher’s [1979: 369–70] suggestion that the possibility of vigilance against distractions, etc., makes the capacity for exerting effort more fairly distributed. Our point here is slightly different, though. We suggest not that such strategies make greater effort possible for an agent who starts out with less, but rather that employment of these strategies is morally praiseworthy *per se*, even if they reduce the need to exert effort.
